# The functions of apolipoproteins and lipoproteins in health and disease

**DOI:** 10.1186/s43556-024-00218-7

**Published:** 2024-10-28

**Authors:** Zijun Ma, Jixin Zhong, Wei Tu, Shiliang Li, Jun Chen

**Affiliations:** 1 Sinopharm Dongfeng General Hospital (Hubei Clinical Research Center of Hypertension), Hubei Key Laboratory of Wudang Local Chinese Medicine Research, Hubei University of Medicine, Shiyan, China; 2 Division of Cardiothoracic and Vascular Surgery, Tongji Hospital, Tongji Medical College, Huazhong University of Science and Technology, Wuhan, China; 3 Department of Rheumatology and Immunology, Tongji Hospital, Huazhong University of Science and Technology, Wuhan, 430030 Hubei China; 4 Key Laboratory of Vascular Aging (HUST), Ministry of Education, Wuhan, 430030 Hubei China

**Keywords:** Lipoproteins, Apolipoproteins, Lp(a), Cardiovascular disease, Therapeutic target

## Abstract

Lipoproteins and apolipoproteins are crucial in lipid metabolism, functioning as essential mediators in the transport of cholesterol and triglycerides and being closely related to the pathogenesis of multiple systems, including cardiovascular. Lipoproteins a (Lp(a)), as a unique subclass of lipoproteins, is a low-density lipoprotein(LDL)-like particle with pro-atherosclerotic and pro-inflammatory properties, displaying high heritability. More and more strong evidence points to a possible link between high amounts of Lp(a) and cardiac conditions like atherosclerotic cardiovascular disease (ASCVD) and aortic stenosis (AS), making it a risk factor for heart diseases. In recent years, Lp(a)'s role in other diseases, including neurological disorders and cancer, has been increasingly recognized. Although therapies aimed at low-density lipoprotein cholesterol (LDL-C) and high-density lipoprotein cholesterol (HDL-C) have achieved significant success, elevated Lp(a) levels remain a significant clinical management problem. Despite the limited efficacy of current lipid-lowering therapies, major clinical advances in new Lp(a)-lowering therapies have significantly advanced the field. This review, grounded in the pathophysiology of lipoproteins, seeks to summarize the wide-ranging connections between lipoproteins (such as LDL-C and HDL-C) and various diseases, alongside the latest clinical developments, special emphasis is placed on the pivotal role of Lp(a) in cardiovascular disease, while also examining its future potential and mechanisms in other conditions. Furthermore, this review discusses Lp(a)-lowering therapies and highlights significant recent advances in emerging treatments, advocates for further exploration into Lp(a)'s pathogenic mechanisms and its potential as a therapeutic target, proposing new secondary prevention strategies for high-risk individuals.

## Introduction

Lipoproteins and apolipoproteins are essential in maintaining lipid metabolic balance in the human body. As complexes of lipids and proteins, lipoproteins participate in lipid transport and regulate cell function and signaling [1–3]. Depending on their density, size, and function, lipoproteins are divided into several subtypes, including chylomicrons (CM), high-density lipoproteins (HDL), and low-density lipoproteins (LDL), each playing an essential role in maintaining metabolic balance and regulating cholesterol transport [4–6]. Apolipoproteins (ApoA, ApoB), key components of lipoprotein structure and function, are crucial for lipoprotein stability, receptor recognition, and metabolic regulation [7]. HDL-cholesterol (HDL-C) is commonly thought to have anti-atherosclerotic characteristics because of its involvement in facilitating reverse cholesterol transport [8], whereas increased LDL-C is a critical risk factor for several atherosclerotic illnesses [9].

Lipoprotein(a) (Lp(a)) has increasingly become a research focal point in recent years because of its distinctive biological properties. While Lp(a) has a lipid core resembling that of LDL, it also has a distinct apolipoprotein(a) [Apo(a)], which confers upon it particular biological properties. According to available genetic and clinical data, Lp(a) is a separate hereditary risk factor for aortic stenosis (AS) and atherosclerotic cardiovascular disease (ASCVD). In their 2022 Lp(a) consensus statement, the European Atherosclerosis Society and the Canadian Cardiovascular Society emphasized the importance of measuring Lp(a) as part of a thorough assessment of ASCVD risk. They also recommended that all adults have at least one measurement of Lp(a) throughout their lives [10–12]. Not limited to cardiovascular diseases, the relationship between Lp(a) levels and other systemic conditions has also attracted attention in recent years. Furthermore, despite the availability of effective interventions for many lipid-related cardiovascular risk factors, treatments targeting Lp(a) have had limited success, emerging therapies offer hope for a new golden age in treatment.

Thus, although many lipoproteins and apolipoproteins are significant in cardiovascular and metabolic diseases, this review will focus on Lp(a). Based on the pathophysiology and clinical trials of different lipoprotein subtypes, this review systematically examines their distinct biological roles in disease development and progression, alongside their interactions with other lipoproteins and apolipoproteins, aiming to present the latest clinical advances concerning their roles in cardiovascular and other diseases, with a focus on Lp(a)-lowering therapies and recent breakthroughs in emerging treatments, while exploring the future potential and mechanisms of Lp(a) in cardiovascular diseases (beyond ASCVD and AS) and other diseases.

## The structure, genetic inheritance, and pathophysiology of apolipoproteins and lipoproteins

### Common apolipoproteins and lipoproteins

**Fig. 1:**
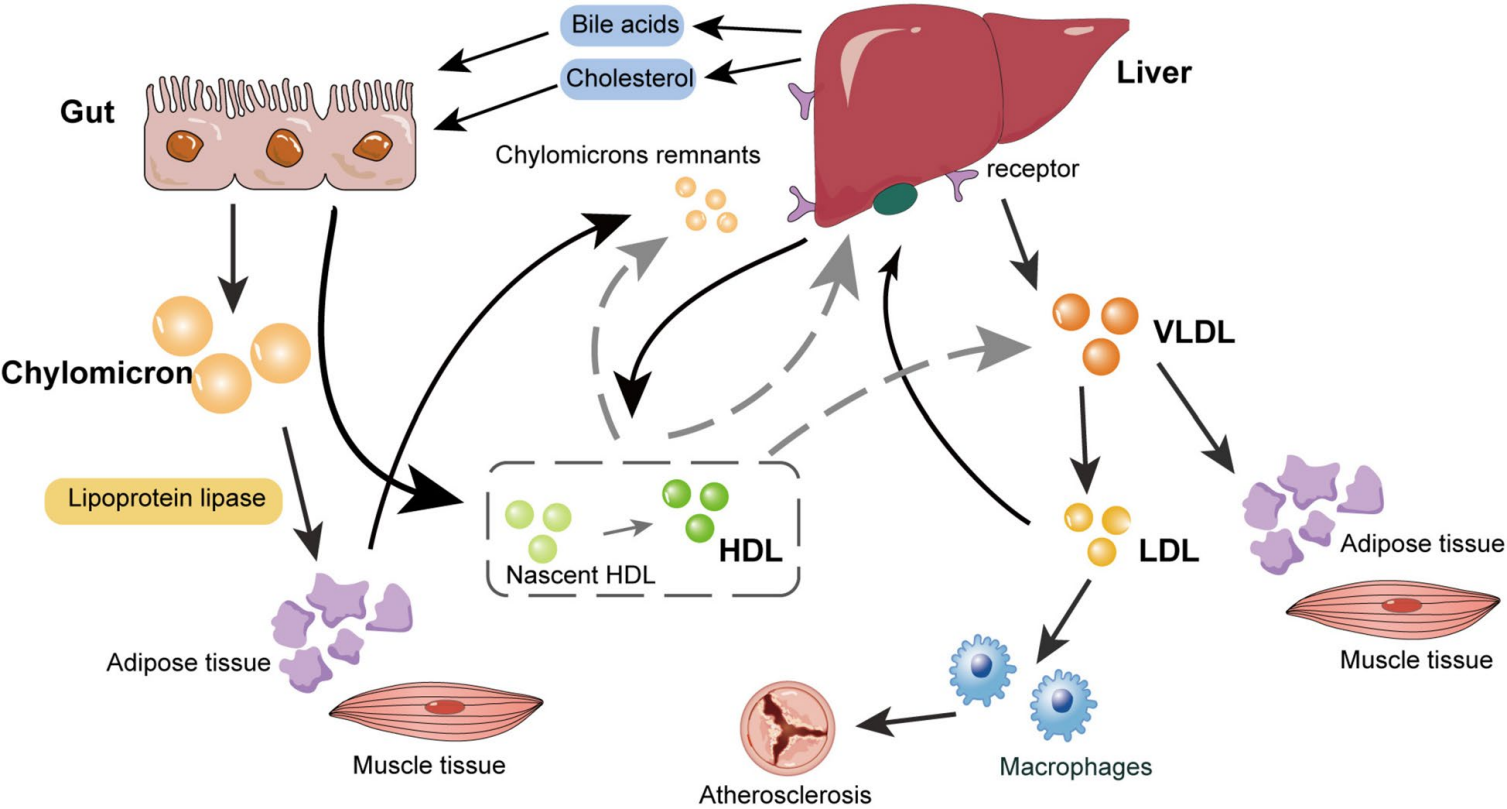
Illustrative diagram of lipid transport and metabolic pathways. The figure shows the lipid processing pathways through the gut, liver and peripheral tissues. Chylomicrons from the diet are formed in the epithelial cells of the small intestine, and after being broken down by lipoprotein lipase, they transport dietary triglycerides and cholesterol to adipose and muscle tissues. VLDL, synthesized in the liver, carries triglycerides that are progressively hydrolyzed to become LDL, while HDL promotes the reverse transport of cholesterol back to the liver. HDL, High-density lipoprotein; VLDL, very-low-density lipoprotein; LDL, low-density lipoprotein

Apo, acting as lipid carriers, are attached to the surface of lipoprotein particles, providing recognition sites for cell membrane receptors and serving as structural support and functional regulators [13].  Different apo have distinct roles in lipid metabolism,for instance, apolipoprotein B (apo(B)), especially ApoB100, is the primary apolipoprotein in LDL, playing a role in LDL-C metabolism and binding to LDL receptors [14]. Apolipoprotein E (ApoE) is primarily present in HDL and remnant particles, while apolipoprotein A1 (ApoA1) is the main component of HDL [15]. Various apolipoproteins bind with lipids to form lipoproteins of different densities, lipoproteins are spherical entities composed of hydrophobic lipid cores and hydrophilic exteriors, responsible for lipid molecule transport [16]. Based on size and density, lipoproteins are conventionally classified into CM, very LDL (VLDL), LDL, and HDL ( \* MERGEFORMAT Fig. 1).

The levels of most apolipoproteins and lipoproteins are regulated by various genes, which control not only the synthesis, secretion, and metabolism of apolipoproteins but also the size, number, and metabolic pathways of lipoprotein particles in the body. For instance, the APOB gene dictates the synthesis of apo(B), impacting the formation of LDL and VLDL and thus increasing the risk of atherosclerosis [17].  The ApoA1 gene encodes the principal apolipoprotein in HDL, directly affecting reverse cholesterol transport and its anti-atherosclerotic potential [18]. The APOE gene regulates the removal of lipoprotein remnant particles by encoding ApoE [19, 20].

In recent years, substantial evidence has indicated that apolipoproteins and lipoproteins are crucial in inflammation, thrombosis, and atherosclerosis. In particular, LDL buildup in the arterial wall causes a localized inflammatory response, leading to the formation of arterial plaques and the narrowing of blood vessels. When atherosclerotic plaques rupture, this induces thrombosis and increases the risk of cardiovascular events [21, 22]. Concurrently, HDL assumes a similarly significant function in this process. Research indicates that diminished HDL levels may correlate with decreased cholesterol outflow from foam cells, consequently worsening atherosclerosis [23]. HDL not only removes excess cholesterol through a reverse cholesterol transport mechanism, but also has anti-inflammatory and antioxidant properties that inhibit the development of atherosclerosis. In addition, apolipoproteins (e.g., apoB and apoE) play key roles in these pathologic processes. ApoB is a major component of LDL and its associated particles and is directly involved in cholesterol metabolism [14], whereas apoE plays an important scavenging role primarily in HDL and residual particles [19]. In conclusion, apolipoproteins and lipoproteins not only play important roles in lipid metabolism, but they also show complex interactions in pathological processes such as inflammatory responses and atherosclerosis, providing new insights into the pathogenesis of cardiovascular diseases.

### Specific lipoproteins (Lp(a))

**Fig. 2:**
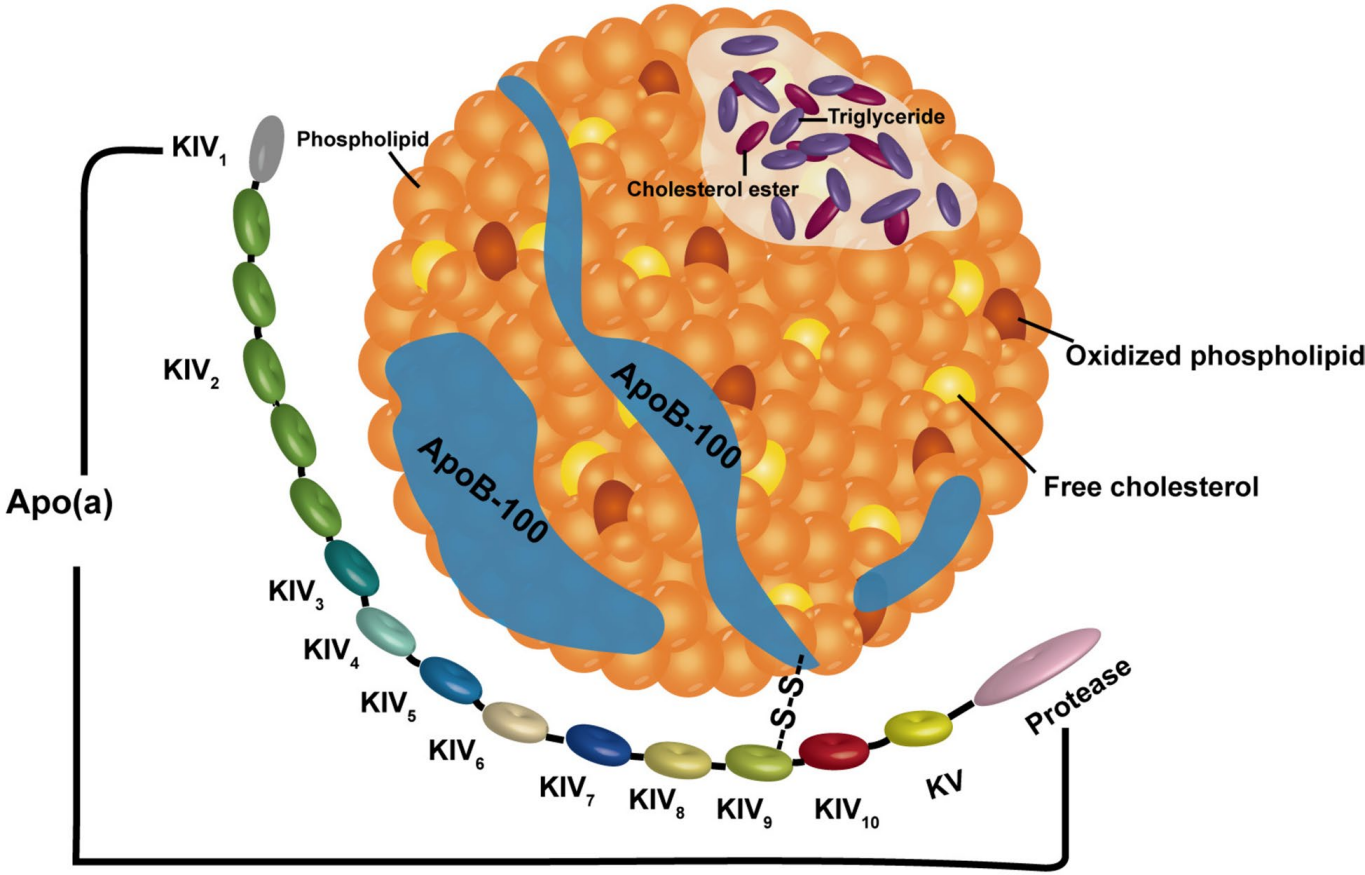
Illustration of the structure of Lp(a). Lp(a) mainly consists of apoB-100 protein, a lipid core (triglycerides, cholesterol esters, free cholesterol), and Apo(a).Apo(a) is encoded by the LPA gene, which is highly homologous to the plasminogen gene, and contains a Kringle (K) IV domain (KIV 1–KIV 10), a Kringle V (KV) domain, and an inactive protease domain. Lp(a), Lipoprotein(a); Apo(a), Apolipoprotein(a); KIV, kringle IV; KV, kringle V

In addition to standard classifications, Lp(a) is a distinct type of lipoprotein, with a unique structure that differentiates it from other lipoproteins. Lp(a) is composed of lipoprotein particles that share structural similarities with LDL in their protein and lipid composition. Both Lp(a) and LDL contain apolipoprotein B100 (apo-B100) [21]. On the other hand, Lp(a) is characterized by the presence of a specifically hydrophilic and highly glycosylated apolipoprotein (a) [apo(a)] that is covalently attached to the apo-B100 molecule by means of a single disulphide bond [22–24]. Apo(a), which is encoded by the LPA gene, shows a striking resemblance to the PLG gene that produces plasminogen. The structure of this features a plasminogen (Kringle) (K) domain IV (KIV 1—KIV 10), a Kringle domain V (KV), and an inactive protease domain (P). The LPA gene has evolved through duplication and modification of the plasminogen gene. The significant variability of apo(a) is intricately linked to the structural domain of the protein known as "kringle" (K), a crucial component of the plasminogen gene. Typically, each KIV type is represented as a individual replica. However, KIV-2 isoforms are an exception to this rule, as they can have diverse numbers of replicas of the same types,, ranging from less than 13 to more than 50. The varying number of copies of KIV-2 directly contributes to the size variability of apo(a) [25, 26]. Physically speaking, the lipoprotein fraction of Lp(a) is virtually identical to that of LDL, but the unique presence of apo(a) imparts high heterogeneity to Lp(a)( \* MERGEFORMAT Fig. 2). Lp(a) is synthesized in the liver. Although its catabolism has not been fully elucidated, it is believed that both liver and kidney are involved in its catabolism and clearance [21, 27].

In terms of heritability, Lp(a) is no different, with the LPA gene governing its production and impacting an individual’s Lp(a) levels [28]. However, unlike other lipoproteins, Lp(a)'s genetic influence is not easily modified by lifestyle changes, and conventional lipid-lowering therapies have minimal effects on it. More specifically, plasma concentration of Lp(a) is widely variable among individuals in general population, with its level primarily genetically determined. The LPA gene encodes for the formation of different numbers of KIV2 structural domains, resulting in lipoprotein isoforms of different sizes [29]. Typically, most populations have a negative correlation among apo(a) isozyme size and plasma Lp(a) concentration. In other words, KIV-2 repetitions negatively correlate with Lp(a) concentration. In addition to this, non-genetic factors can also influence Lp(a) levels to some extent, such as kidney function, menopause, physical stress, hormones [30–36], etc.… In overall terms, however, Lp(a) is highly heritable and is not significantly affected by diet, gender, or age [37] ( \* MERGEFORMAT Table 1).

**Table 1:** Effect of non-genetic factors on Lp(a) levels

Factor	The Effects on Lp(a)	Ref
Menopause	Lp(a) levels are usually elevated in women after menopause as estrogen levels decline, possibly related to hormonal changes	[31]
Renal function	Renal insufficiency typically results in markedly elevated Lp(a) levels, particularly in end-stage renal disease	[32]
Hormones	Hormone therapy (e.g., estrogen) may reduce Lp(a) levels in women, whereas testosterone replacement therapy does not have a significant effect in men	[33]
liver function	Lp(a) is synthesized in the liver and its metabolism is impaired in hepatic insufficiency, resulting in elevated levels	[35]
Stress	Chronic physical and psychological stress may indirectly contribute to elevated Lp(a) levels through a pro-inflammatory response	[36]
Diet and Body weight	The direct effects of diet and body weight on Lp(a) are limited, but extreme obesity or malnutrition may alter Lp(a) levels in some cases	[37]

**Fig. 3:**
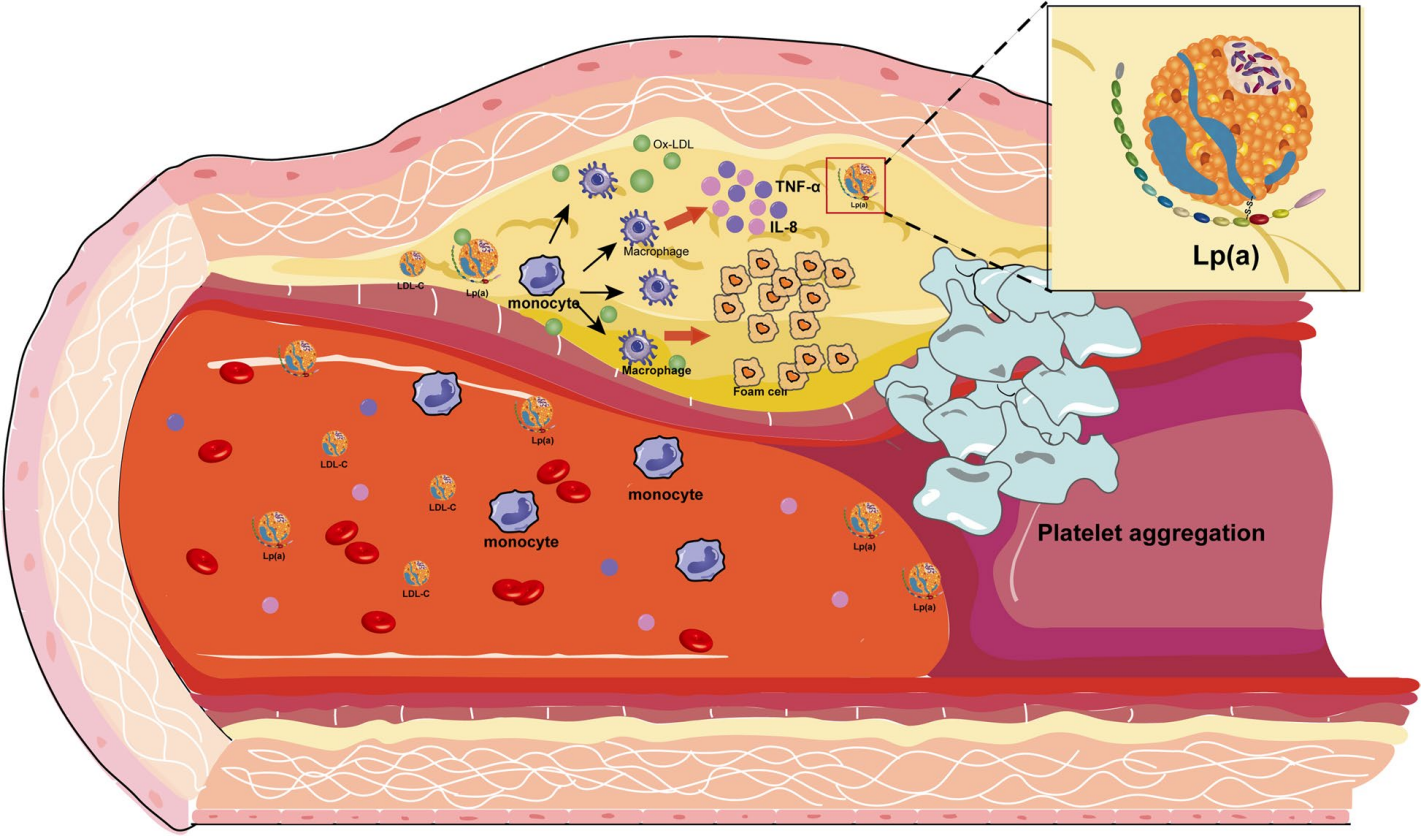
Lp(a) in atherosclerosis progression. This diagram demonstrates how Lp(a) contributes to the formation of atherosclerotic plaques. LDL and Ox-LDL accumulate in the arterial intima, triggering monocyte recruitment and differentiation into macrophages. Macrophages engulf Ox-LDL, forming foam cells and releasing inflammatory cytokines such as TNF-α and IL-6, which promote further plaque development. Lp(a) promotes this process by enhancing monocyte adhesion, oxidative stress, and platelet aggregation, increasing the risk of plaque rupture and thrombosis. Lp(a),Lipoprotein(a);LDL, low-density lipoprotein; Ox-LDL, oxidized low-density lipoprotein

**Fig. 4:**
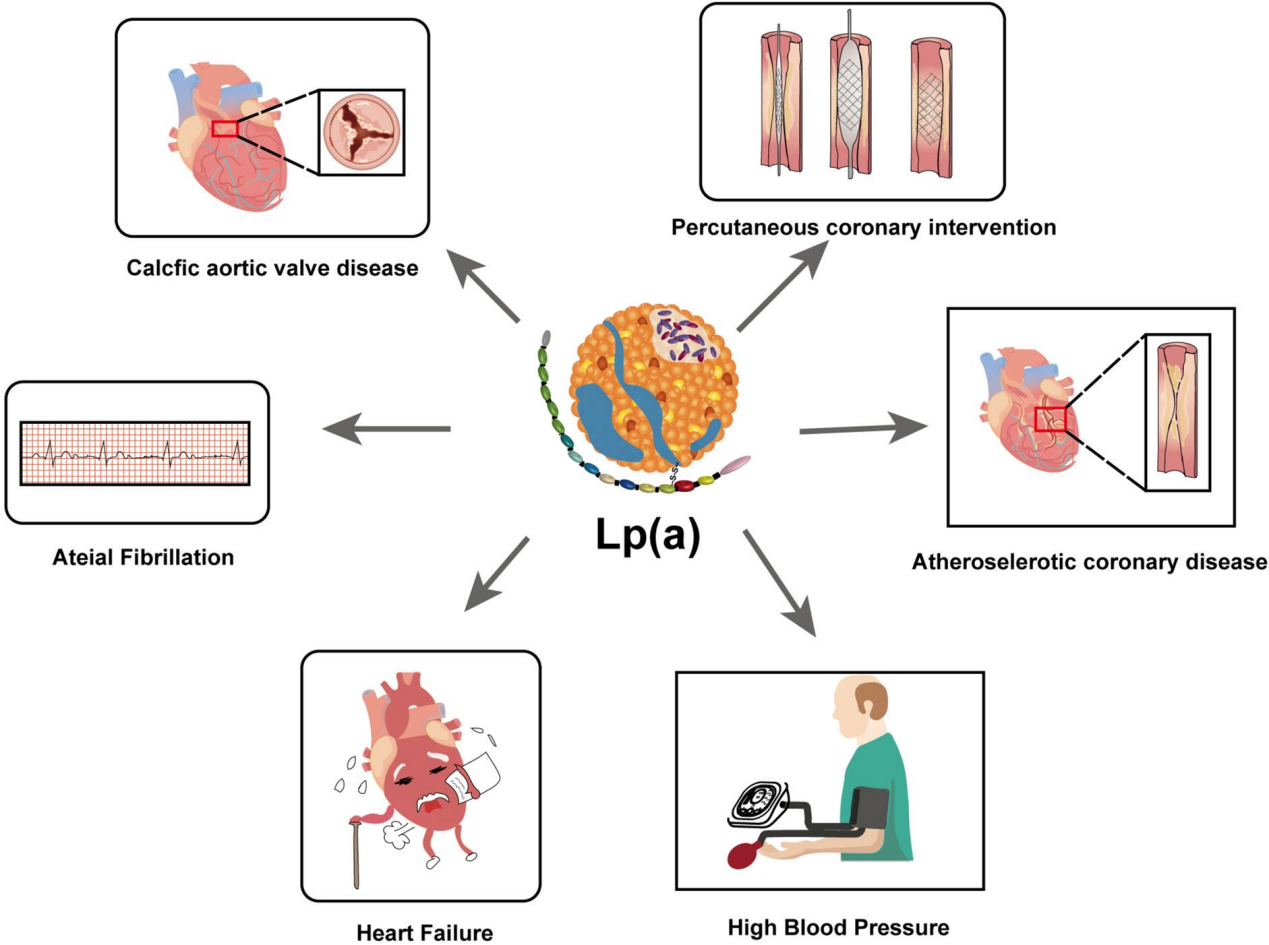
Associations of Lp(a) with Cardiovascular Diseases. This diagram illustrates the role of elevated Lp(a) levels in various cardiovascular conditions. Lp(a) is linked to atherosclerotic coronary disease, calcific aortic valve disease, percutaneous coronary interventions, atrial fibrillation, heart failure, and hypertension. Lp(a), Lipoprotein(a)

However, the normal physiological function of Lp(a) is not clear. Given that apo(a) and fibrinogen exhibit significant homology, and that Lp(a) and LDL share structural similarities, it is hypothesized that Lp(a) may be crucial for the transport of cholesterol, coagulation, and antifibrinolysis [38], but there is a lack of conclusive evidence to reveal its mechanism and effects. High plasma concentrations of Lp(a) are now thought to be associated with atherosclerosis, thrombosis and inflammation (Fig. 3). The structural component of Lp(a) that is oxidised LDL-C (oxLDL-C) has been associated with a variety of atherogenic effects, such as the expansion of vascular smooth muscle cells, the formation of foam cells, and the release of the pro-inflammatory factor IL-8 [39]. Meanwhile, Lp(a) is associated with various pro-thrombotic mechanisms, such as platelet aggregation via thrombin-mediated activation of PAR1 and oxidised phospholipid (OxPL)-mediated activation of CD36 [40], and Lp(a)-induced cytokines promote inflammation, and macrophages are induced by apo(a) to release interleukin-8, monocyte chemotactic proteins, and tumour necrosis factor-alpha (TNF-α) [41] (Fig. 4). As we have stated before, the special structure of Lp(a) gives rise to many potential possibilities for its biological actions, and thus Lp(a) deserves our continued attention as one of the major threats to cardiovascular disease.

## The role of lipoproteins in disease development

**Fig. 5:**
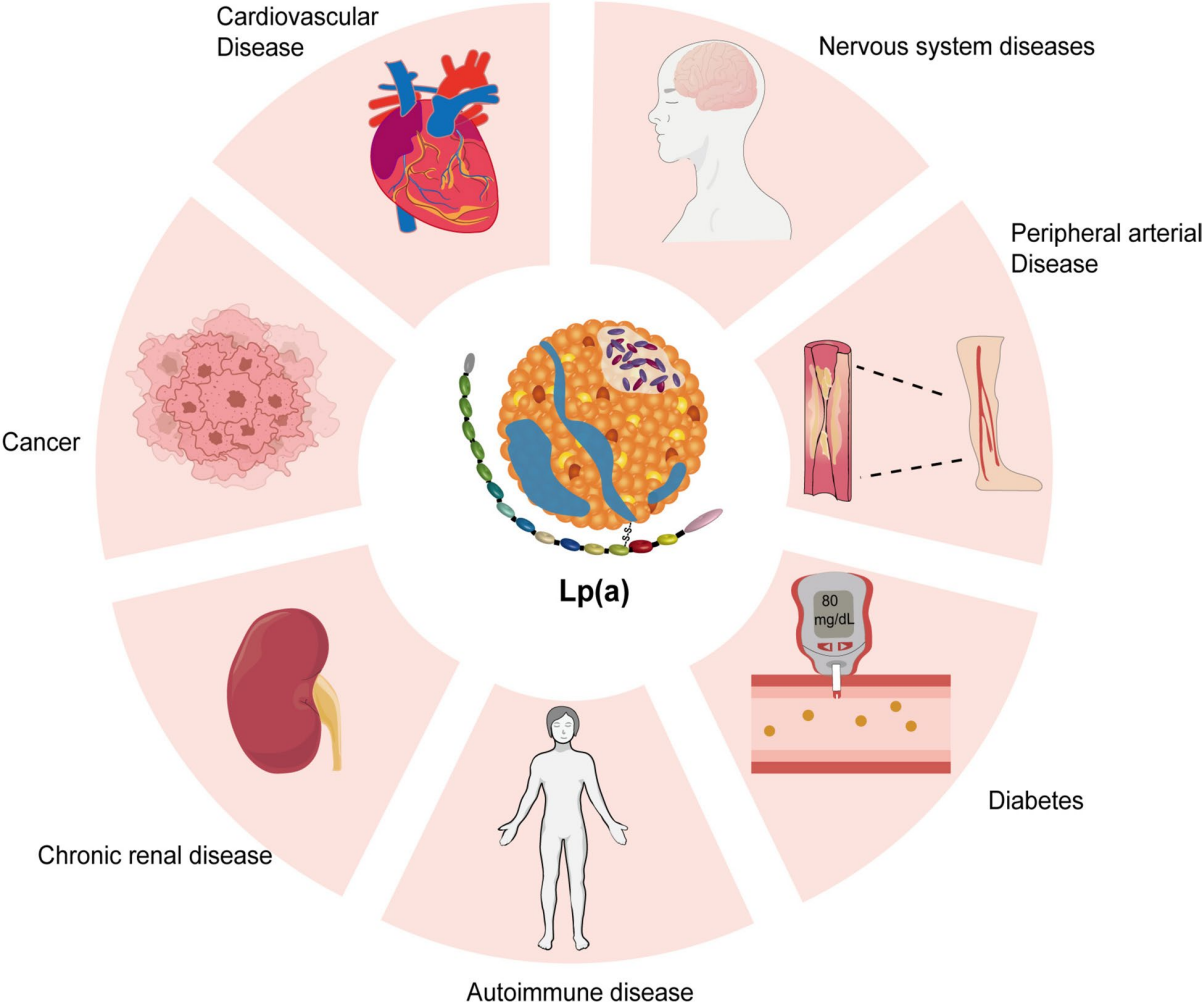
Association of Lp(a) with Various Diseases. This illustration shows the potential links between elevated Lp(a) levels and a range of diseases. High levels of Lp(a) are associated with cardiovascular disease, nervous system diseases, peripheral arterial disease, diabetes, autoimmune diseases, chronic renal disease, and cancer. These associations suggest that Lp(a) plays a role beyond cardiovascular health, impacting multiple organ systems and disease processes. Lp(a), Lipoprotein(a)

It is crucial to acknowledge that lipoproteins are significantly involved in the development of a variety of diseases when discussing the relationship between them and diseases. Various types of lipoproteins, such as LDL-C, HDL-C, and Lp(a), are intimately linked to cardiovascular diseases, peripheral vascular diseases, neurological disorders, cancers, and metabolic-related diseases. Prior research has established the significant roles of LDL-C and HDL-C in various systemic diseases, especially in cardiovascular diseases [42, 43]. Furthermore, the uniqueness of Lp(a) makes it a key biomarker for cardiovascular risk assessment. As previously stated, elevated levels of Lp(a) are strongly related to atherosclerosis, thrombosis, and inflammatory reactions, and its oxidized form plays a key role in developing vascular lesions [39–41]. Therefore, carrying out in-depth research on the functions of lipoproteins—particularly Lp(a)—in various illnesses will facilitate the creation of cutting-edge therapeutic approaches ( \* MERGEFORMAT Fig. 5).

### Lipoproteins and cardiovascular disease

#### Atherosclerotic coronary heart disease

Atherosclerosis is a major pathogenetic mechanism of coronary heart disease (CHD), one of the leading causes of cardiovascular deaths. CHD is characterized by chronic non-decompensated inflammation, lipid accumulation and fibroproliferation [44]. Abnormal lipoproteins are essential for lipid transport and have a major part in the progression of atherosclerosis. Elevated levels of LDL and apo(B) are widely regarded as highly atherogenic and significantly correlated with an higher risk of CHD, while HDL and ApoA1 provide a preventive function by facilitating cholesterol removal [8]. Moreover, numerous studies have demonstrated that Lp(a) is not only an seperate cause of ASCVD but also a significant risk factor for primary and secondary ASCVD prevention [24, 41].

Independent risk factors for CHD are believed to be derived from Lp(a)'s distinct structure, and Lp(a) can contribute to atherosclerotic thrombosis on its own via a variety of mechanisms. Firstly, similar to LDL, Lp(a) promotes atherosclerosis by entering the vessel wall leading to cholesterol deposition, inflammation and calcification [45]. Secondly, Lp(a) particles carry the largest proportion of OxPL among all apo(B)-containing lipoproteins, and phosphorylcholine-containing oxidized phospholipids (OxPL) induce chronic inflammation and are present in large numbers in atherosclerotic lesions [46]. Lastly, although both apo(B) and Lp(a) promote lipid deposition through LDL, the distinctive structure of apo(a) makes Lp(a) more dangerous in terms of plaque rupture and thrombosis, as the structural similarity between apo(a) and plasminogen can inhibit fibrinolysis, influencing the risk of ASCVD [47].

Large-scale clinical trials have demonstrated that elevated plasma LDL-C levels increase the risk of ASCVD [48–50], and this biological impact is closely associated with the level and duration of exposure [51, 52]. Reducing LDL-C levels through lifestyle changes or therapy is critical not only for ASCVD prevention but also for lowering the risk of ASCVD events. HDL-C is thought to be a protective factor against CHD, as opposed to LDL-C [53, 54]. Unfortunately, despite animal model studies showing that functional HDL-C plays a role in limiting atherosclerosis progression and stimulating plaque regression, increasing HDL-C levels in human trials has failed to provide convincing evidence of health benefits [55]. Many epidemiological studies on the lipoprotein of interest, Lp(a), have shown a strong association between elevated plasma Lp(a) concentrations and CHD. Robert Clarke et al. confirmed in the large multicenter PROCARDIS study that differences in LPA are substantially associated with elevated levels of Lp(a) and an increased risk of CHD, supporting the idea that Lp(a) is key to coronary artery disease [56]. Furthermore, multiple extensive clinical studies have demonstrated a strong correlation between Lp(a) levels and the degree of left ventricular hypertrophy as well as the severity of coronary arteries in individuals experiencing fresh myocardial infarctions [57, 58]. Additionally, researchers have discovered that individuals with elevated levels of Lp(a) experience a greater risk of serious cardiovascular events during the average follow-up duration of three years [59], This also applies to those who have experienced a prior myocardial infarction and have elevated levels of Lp(a) [60]. It is worth noting that there are a number of different voices regarding the impact of cardiovascular risk factors. According to the observational data collected by the EPIC-Norfolk study, individuals with lipoprotein (a) concentrations greater than 50 mg/dL who have a lower number of modifiable risk factors, such as smoking, obesity, diabetes, and high blood pressure, have a significantly lower risk of ASCVD events when compared to those who lead unhealthy lifestyles [61]. Recent studies, on the other hand, have shown that patients without a history of ASCVD are independently associated with a greater risk of a first AMI when their Lp(a) levels are high. This remains true irrespective of the existence of any modifiable risk factors, including smoking, hypertension, diabetes, or hyperlipidemia [62].

#### Subclinical coronary artery calcification

The total coronary plaque burden is correlated with coronary calcification, which is a sign of subclinical atherosclerosis. Additionally, the coronary artery calcification (CAC) score is independently associated with ASCVD events [63]. The AHA/ACC guidelines recognize elevated Lp(a) levels (≥ 50 mg/dL or ≥ 125 nmol/L) as cardiovascular risk enhancers and recommend the use of the CAC score as a validated measure to guide decisions regarding primary prevention of ASCVD [64, 65]. It is crucial to note that not all patients with high Lp(a) levels undergo CAC scoring in clinical practice. Likewise, patients with abnormal CAC scores may still carry a high risk of atherosclerosis even if their Lp(a) levels are normal. Therefore, even if asymptomatic coronary calcification is linked to higher Lp(a) levels, the two are considered to be independent risk assessment tools. The utilization of both of these tools together can provide a more comprehensive evaluation for the early detection and treatment of cardiovascular disease. Also, having higher levels of LDL-C is thought to make the progression of coronary calcification more likely. LDL-C promotes coronary calcification directly through oxidation and inflammatory pathways, depositing within the vascular wall to form plaques, which subsequently induce smooth muscle cell calcification and eventually lead to stable calcified plaques [66]. In the meantime, Lp(a) has a more intricate function in this procedure. Experimental findings indicate that Lp(a) can promote calcium deposition in the arterial endothelium by inducing the chondro-osteogenic differentiation of vascular smooth muscle cells (VSMCs) [67]. Moreover, Lp(a) is also a preferential carrier of pro-inflammatory factors such as oxidized lipids, capable of inducing oxidative stress in endothelial cells and further promoting VSMC chondro-osteogenic differentiation [68].

Current clinical research has clearly demonstrated the strong link between LDL-C and CAC, particularly in individuals with chronically elevated LDL levels, where the risk of CAC progression is significantly increased [69, 70], and statin therapy has been demonstrated to decrease the volume of non-calcified plaques in addition to lowering LDL-C levels [71]. In comparison, the role of Lp(a) has attracted more attention in recent years. A large cohort study by Parveen K. Garg et al. included 6705 people with atherosclerosis and assessed changes in CAC in people with high and low concentrations of Lp(a) [72]. Research indicates that the likelihood of CAC progression is roughly 67% greater in multiracial populations exhibiting higher Lp(a) levels compared to those with diminished Lp(a) concentrations [72]. A recent meta-analysis indicated that elevated Lp(a) levels over time in asymptomatic CVD populations may accelerate CAC progression, eventually leading to cardiovascular events. Patients with asymptomatic cardiovascular disease who are at risk of CAC are identified by elevated Lp(a) levels, which are recognized as an independent marker [73]. The close interaction between elevated Lp(a) levels and CAC suggests that drugs aimed at lowering Lp(a) could be a novel and promising therapeutic strategy for the primary prevention of ASCVD.

#### Calcific aortic valve disease

Since no medication has been demonstrated to either slow down or stop the course of the disease, therapy of aortic valve calcification (AVS) and calcific aortic stenosis (CAVS) remains difficult to date. For severe aortis stenosis or symptomatic individuals, surgery or transcatheter valve implantation is the only viable therapy option. Similar to the mechanism underlying coronary calcification, lipid deposition, inflammatory cell infiltration, extracellular matrix dysregulation, and the osteogenic transformation of valvular interstitial cells contribute to the pathophysiology of this disease [74]. While LDL-C can accelerate calcification through pathways such as oxidation, inflammation, and lipid deposition and is somewhat associated with AVS [75], in contrast, the link between Lp(a) and CAVS is much stronger and clearer [76]. European Atherosclerosis Society Consensus Statement 2022 updates evidence for role of Lp(a) in ASCVD and A S, affirming its strong association with in AS [10].

Initial studies demonstrated a notable relationship among increased Lp(a) levels and aortic valve disease [77]. However, it was initially uncertain whether Lp(a) was a direct causative factor of the disease or merely associated with increased disease risk. Multiple genetic and epidemiological studies conducted since 2013 have shown that variations at the LPA gene locus, which determines Lp(a) levels genetically and is unaffected by aortic valve disease, significantly increase the risk of developing AVS [78]. This implies that rather than being a result condition of the illness, increased Lp(a) is a major causative factor in AVS, and this relationship is consistent across different populations. The crucial and causative role of Lp(a) in AVS among the general populace has been well-established, a study combining two extended analyses of the Danish general population (*n* = 77,680), In individuals with higher Lp(a) concentrations, the risk of AVS increased in a dose-dependent manner, as demonstrated by Pia R. Kamstrup et al. [79]. A significant portion of people with familial hypercholesterolemia had elevated Lp(a) levels in observations [80], and these higher levels indicate an intensified potential risk as they are positively linked to higher risks of cardiovascular disease [81, 82]. Simultaneously, increased levels of Lp(a) are not only linked to the beginning of a disease, but can also accelerate the advancement of aortic stenosis, ultimately resulting in premature replacement of the aortic valve or death [83, 84]. It is noteworthy that the incidence of aortic valve malformations is higher in males compared with females, and that this phenomenon is also seen in older and younger individuals. The stronger association of Lp(a) with aortic valve disease in males and younger individuals may indicate that Lp(a) plays a unique role in the initiation of the disease, and more in-depth studies are needed to explore whether and how gender and age interfere with the effects of Lp(a) on AVS.

#### PCI (percutaneous coronary intervention)

Effective management of lipid levels post-PCI can significantly improve the long-term prognosis of CHD patients. Research indicates that high LDL-C levels are linked to increased late cardiovascular event rates after PCI [85], and early intensive statin therapy can reduce perioperative cardiovascular events while improving long-term prognosis and lowering the risk of long-term cardiovascular events [86, 87]. The ESC guidelines suggest that acute coronary syndrome patients undergoing PCI should consider high-dose statin therapy for long-term lipid-lowering to achieve the recommended target levels [88].

To date, Lp(a) has been used more often to study the relationship with atherosclerotic cardiovascular disease, but less research has been done on the role of Lp(a) and long-term clinical prognosis in post-PCI patients. Satoru Suwa et al. were the first to propose that Lp(a) levels are linked to long-term adverse clinical outcomes in coronary artery disease(CAD) patients who are receiving statin therapy following PCI [89]. A study examining the predictive significance of initial levels of acute temporal reactants following PCI revealed that patients who experienced MACE had notably elevated Lp(a) levels compared to those who did not have MACE [90]. Meanwhile, HuiHui Liu et al. discovered that high lipoprotein(a) levels may be related with poor prognosis after PCI in patients with stable CAD within a substantial cohort with long-term follow-up [91]. Regarding Lp(a)'s impact on long-term cardiovascular results in PCI patients, there is disagreement. Following up on 6714 patients after PCI procedures showed that Lp(a) is a reliable indicator of the development of complex coronary artery disease in PCI patients, yet it is not linked to their long-term cardiovascular outcomes [92]. These results may be the result of conflicting multifactorial factors, such as race, duration of follow-up, gender, pre-PCI status (stable CVD or acute myocardial infarction, etc.), and method of Lp(a) measurement, Therefore, we need more long-term large-sample clinical trials to further explore the effect of Lp(a) on long-term prognosis after PCI.

#### Heart failure

New consensus statement on lipoprotein(a) published by EAS in 2022 suggests differential association of Lp(a) with different cardiovascular outcomes, According to the available data, myocardial infarction and aortic stenosis had the highest correlation, followed by heart failure [93]. In a thorough study involving the whole Danish population, Kamstrup et al. found a clear link between higher risk of heart failure and elevated levels of Lp(a). The risk steadily grew as lipoprotein (a) levels above 90%, with a 1.6- to 1.8-fold increase in risk [94]. A number of studies in recent years have confirmed this view and have suggested that baseline hs-CRP levels, racial differences, gender, individual factors such as having DM or CHD, and medication use may alter the link between serum Lp(a) levels and study endpoints [95–98].

In addition, numerous studies have shown that the incidence of MACEs in heart failure patients with atherosclerosis can be significantly reduced by lowering LDL-C levels for other lipoproteins, such as LDL [99, 100]. However, some research indicates that in people with severe heart failure, extremely low LDL-C levels are linked to poorer prognosis [101]. To date, the relationship between lipoproteins (Lp(a) and LDL-C) and heart failure can be explained by several mechanisms: First, the development of heart failure is closely linked to myocardial infarction, aortic valve stenosis, and hypertension. Elevated Lp(a) and LDL levels are partially explained by their strong correlation with aortic stenosis and myocardial infarction, which increases the risk of heart failure [102]; Second, the atherogenic properties of Lp(a) and LDL-C itself result in increased stiffness of the arterial walls, raising the cardiac afterload and thus heightening the risk of heart failure [103]. In addition, unknown factors, such as inflammation, may make additional contributions to unknown mechanisms independent of ischemic heart disease [104]. Heart failure is a strongly complex and heterogeneous clinical syndrome that represents an advanced stage of cardiovascular disease, and we look forward to future studies focusing on the association between Lp(a) and LDL-C and the different clinical conditions associated with heart failure. In the meantime, future studies targeting specific treatments to reduce Lp(a) and LDL-C should include HF as one of the endpoints.

#### Atrial fibrillation

Lipoproteins are very important in the development of atrial fibrillation (AF), which is commonly linked to structural changes in the heart, hemodynamic abnormalities, and inflammation [105, 106]. LDL-C may contribute to the onset of AF by exacerbating atherosclerosis, causing myocardial ischemia and structural heart changes (e.g., left atrial enlargement or fibrosis), and the ARIC study demonstrated a significant correlation among high LDL-C levels and AF occurrence [107]. However, due to its anti-inflammatory and antioxidant characteristics, HDL-C reduces the incidence of atrial fibrillation (AF) and is strongly linked to new-onset AF [108].

Several studies are currently exploring the relationship between Lp(a) and atrial fibrillation (AF). In previous published studies, we have heard two different voices. There are those who argue that while Lp(a) is associated with diseases where atherosclerosis is the underlying mechanism, AF is not associated with Lp(a) and that there is no meaningful linear relationship between high levels of Lp(a) and AF [109, 110], and no association was found between Lp(a) levels and the incidence of AF in a large cohort of long-term follow-up [111]. Ironically, a large Chinese study found that patients who had or did not have AF had significantly lower Lp(a) levels (this link was significant only in women) [112]. Those whose Lp(a) levels were 30 mg/dL or higher had a lower risk of atherosclerosis fibrillation (AF) than those whose levels were normal, according to the Multi-Ethnic Study of Atherosclerosis [113]. On the other hand, higher Lp(a) levels were associated with a higher risk of developing AF, according to an epidemiologic and genetic analysis of the UK Biobank carried out by Pedrum Mohammadi-Shemirani et al. [114], they hypothesized that Lp(a) influences myocardial tissue beyond the aortic valve and coronary arteries, and this finding was replicated in two Mendelian randomization analyses that utilized independent data [115, 116]. It is clear that the relationship between Lp(a) and AF cannot be definitively concluded at this time, and more and better evidence must be obtained to fully understand and assess the association between Lp(a) and AF and the underlying mechanisms of action, including whether the effect of Lp(a) on AF is independent of the effects of ASCVD, gender and ethnicity, the mechanisms of atherosclerosis and inflammation, and whether Lp(a) affects myocardial tissues other than aortic valves and coronary arteries.

#### High blood pressure

A significant amount of evidence suggests that hypertension is the primary risk factor for ischemic cardiac disease, stroke, and other cardiovascular conditions [117–119]. There is a complex and multifactorial pathophysiology involved here. For example, vascular endothelial dysfunction and dyslipidemia greatly influence the morbidity and mortality of hypertension. From a mechanistic perspective, Lp(a) and LDL play similar roles in the development of hypertension. They are closely linked to the occurrence of hypertension, with one of the potential contributing factors being their direct impact on the vascular wall and their reliance on renal clearance, which may promote hypertension [120]. According to in vitro research, high levels of Lp(a) can cause endothelial and vascular smooth muscle cell dysfunction as well as directly cause atherosclerosis [121], which is similar to the effects of LDL-C [48]. In children, healthy adolescents, and adults with familial hypercholesterolemia, elevated Lp(a) concentrations have been linked to endothelial dysfunction, according to preliminary research [122, 123]. Furthermore, there is a strong correlation between Lp(a) and LDL-C and an elevated risk of hypertension-related complications, which is not limited to the development of hypertension. Leonardo A Sechi et al., in a cross-sectional research of individuals with mild to moderate hypertension, found that serum Lp(a) was a sensitive indicator of the severity of target organ injury in patients with essential hypertension [124]. Elevated LDL-C or non-HDL-C levels in the elderly further increase the risk of heart disease in people with normal high blood pressure, according to the Cardiovascular Health Study (CHS) [125]. A recent large-scale multiethnic cohort study of individuals who did not have ASCVD clinically at baseline, which evaluated the combined effects of Lp(a) and hypertension on incident ASCVD, elevated Lp(a) significantly altered the relationship among hypertension and cardiovascular disease, and the Lp(a)-related risk of cardiovascular disease was not significant in patients without hypertension [126]. Similarly, this phenomenon is also apparent in patients who have been diagnosed with CAD [127]. These findings suggest that Lp(a) may require specific pathophysiologic (e.g., endothelial damage) interactions associated with hypertension to fulfill its atherosclerotic and inflammatory potential [128]. Of course, more convincing evidence is needed to validate this conjecture, and elevated Lp(a) levels in hypertensive patients may serve as a wake-up call to clinicians that therapies aimed at lowering Lp(a) may be clinically crucial for the prevention and therapy of hypertension and its target organ damage to prevent cardiovascular events.

### Lipoproteins and peripheral arterial disease (PAD)

PAD is marked by atherosclerosis in the arteries of the lower limbs, it ranks as the third most prevalent atherosclerotic vascular condition, following CAD and stroke [129]. Research indicates that PAD considerably raises mortality, with patients exhibiting symptomatic PAD having more than double the risk of death, and the risk rises with the progression of PAD severity [130]. Studies show a strong correlation between LDL-C and HDL-C and the onset and progression of peripheral artery disease, with persistently high LDL-C levels acting as a stand-alone risk factor for the development of this illness in new cases [131]. In addition, compared to LDL-C, HDL-related lipid metrics show a stronger connection to PAD, making them better therapeutic targets for PAD prevention [132]. Nonetheless, Lp(a) is now recognized for its significant contribution to PAD, alongside LDL-C and HDL-C. Because of its unique effects on blood vessels that are pro-inflammatory, pro-coagulant, and calcification-promoting, Lp(a) not only accelerates the development of atherosclerosis but is also closely associated with peripheral vascular blockages and ischemic symptoms in patients with PAD [133].

Growing amounts of clinical data have demonstrated the significance of Lp(a) in PAD in recent years. An extensive prospective study that examined the relationship between Lp(a) and PAD in the Copenhagen cohort found that people whose Lp(a) levels were in the 99th percentile or higher had a considerably elevated risk of PAD, as well as a higher incidence of major adverse limb events (MALE) and lower limb amputations compared to those whose levels were in the 50th percentile or lower [134]. Additional research has supported this conclusion, as Small et al. analyzed data from three different cohorts, showing that higher Lp(a) levels were linked to PAD, independent of inflammation levels in both primary and secondary prevention populations [135]. In patients with advanced PAD, Verwer et al. found that Lp(a) could be a useful risk stratification tool for predicting future MALEs after a median follow-up duration of 5.6 years [133]. Then, regardless of LDL-C levels and statin therapy, a study comprising 1,169 patients with symptomatic peripheral artery disease (PAD) who underwent successful endovascular treatment demonstrated that Lp(a) remained a standalone indicator of MALE and major adverse cardiovascular events (MACE) [136]. In the field of genetics, genome-wide association studies (GWAS) have highlighted a strong link between the LPA gene and PAD, indicating that therapeutic modulation of circulating Lp(a) could be effective for the PAD phenotype [137]. A recent study evaluating the causal relationship between plasma biomarkers and peripheral artery disease further supports this idea [138]. In summary, various studies in recent years have provided strong evidence for the role of Lp(a) in PAD, indicating its substantial potential as a novel therapeutic target for PAD. While PCSK9 inhibitors have demonstrated efficacy in lowering serum Lp(a) levels and mitigating adverse events associated with PAD, there is a dearth of research on the management of PAD individuals who have increased Lp(a) and insufficient evidence for Lp(a)-specific therapies at this time. Future research should focus on whether more aggressive reduction of Lp(a) levels in PAD populations is necessary.

### Lipoproteins and neurological diseases

As one of the global public health issues, stroke ranks second among causes of death. According to statistics, 1 in 4 individuals over the age of 25 will experience a stroke in their lifetime, and its incidence, mortality, and disability rates are on the rise [139]. Lipoproteins are thoroughly examined risk factor for atherosclerosis, and the correlation among LDL-C and stroke is extensively proven, with LDL-C being the most reliably predicted lipid marker for stroke [140]. Recent guidelines advocate lowering LDL-C to prevent stroke and its recurrence [88, 141]. Although some studies in recent years suggested that aggressive LDL-C reduction might increase the risk of hemorrhagic stroke, cognitive impairment, or dementia, a recent statement has denied this claim, stating that the benefits of LDL-C reduction far exceed the possible risks [140]. The results of studies on Lp(a) and stroke have not been entirely consistent, but recent research suggests a positive correlation between cerebrovascular events, particularly ischemic stroke incidence or recurrence, and elevated serum Lp(a) levels [142–144]. Nevertheless, a recent Mendelian study identified a strong correlation between higher Lp(a) levels and a heightened risk of large-artery stroke and a decreased risk of small-vessel stroke [145]. Like LDL, Lp(a) molecules can deposit directly onto the arterial walls, promoting foam cell formation and contributing to atherosclerosis. Moreover, Lp(a) raises stroke risk through pro-thrombotic and pro-inflammatory pathways [146]. Nevertheless, the precise mechanisms underlying Lp(a)-mediated stroke risk elevation remain uncertain, warranting further investigation in future studies. Reducing total ASCVD risk has become the main objective of stroke patient care in recent years. A recent study demonstrated that high Lp(a) levels are linked to stroke risk in primary and secondary prevention populations, irrespective of baseline hs-CRP values [135]. Future research is required to investigate whether therapies aimed at lowering Lp(a) can further reduce stroke risk.

In addition to stroke, cognitive impairment is thought to be possibly caused by atherosclerotic effects associated with alterations in lipid profiles, research has identified a correlation between plasma lipid levels (LDL-C, HDL-C, and Lp(a)) and cognitive function [147]. Alzheimer's disease, a progressive neurodegenerative disorder, has no known cure and, together with vascular dementia, is considered a major form of dementia that affects the elderly [148]. Earlier research discovered that increased Lp(a) levels are linked to the risk of Alzheimer's disease and vascular dementia, and shifts in Lp(a) may help detect vascular damage in Alzheimer's disease or vascular dementia [149]. Nonetheless, there is little data to support the view that lowering Lp(a) levels improves the prognosis of Alzheimer's disease or vascular dementia, emphasizing the need for additional research.

Depression (also called depressive disorder) is a common mental disorder, characterized by prolonged periods of low mood, loss of pleasure, or loss of interest in activities. Research suggests a negative correlation between depression and low LDL-C levels, which might be due to changes in the structure and function of central nervous terminals mediated by cholesterol depletion [150]. Additionally, research has shown that patients with depression exhibit significantly higher Lp(a) levels compared to healthy populations, and these levels are correlated with the worsening of newly onset depressive symptoms [151]. In particular, Lp(a) is thought to be a potential biomarker for anhedonia in male patients with unipolar and bipolar depression, although the precise mechanisms remain unclear, it is hypothesized that they may involve complex interactions among immune-inflammatory responses, lipoprotein profiles, and mood disorders [152]. In terms of treatment, some studies suggest that lipoprotein apheresis could raise the risk of depression [153], so careful monitoring of the mental status of patients undergoing this therapy is recommended.

### Lipoproteins and *cancer*

The incidence of cancer is still rising and is predicted to increase by more than 61% by 2040. Cancer is the second biggest cause of death worldwide, despite the introduction of innovative therapeutic approaches and predicting technologies for various tumors [154]. In recent studies, the role of lipoproteins in cancer development, progression, malignant tumor formation and metastasis has gradually emerged. For example, studies have shown a direct correlation between LDL-C and breast cancer risk, with breast cancer patients with higher plasma LDL-C levels tending to be accompanied by larger tumor volumes and a higher probability of lymph node metastasis [155]. In addition, elevated LDL-C levels are also strongly associated with colorectal cancer incidence, cancer cell migration, and poor prognosis [156–158], as well as with prostate and pancreatic cancer prevalence, tumor stage, and the risk of drug resistance [159]. elevated LDL-C may enhance metastasis by supporting tumor growth and proliferation, and by facilitating the evasion of immune surveillance by tumor cells.

Levels of HDL-C, in contrast to those of LDL-C, are strongly and adversely linked to the risk of numerous malignancies. A strong correlation was observed between cancer mortality in women and low HDL-C levels [160]. Higher blood HDL-C levels have also been linked to a decreased risk of tumor recurrence and death in the majority of tumor types, according to multiple meta-analyses [36, 161, 162]. In addition, elevated HDL-C was positively associated with improved disease-free survival and overall survival, which may be related to the antitumor activity of HDL and its components to some extent. However, although the roles of LDL and HDL in cancer are becoming clearer, their specific mechanisms need to be further explored. Subsequent research will enhance our comprehension of the function of lipoproteins in cancer progression.

In recent years, the role of Lp(a) in cancer as a highly publicized biomarker has gradually attracted widespread attention. In breast cancer patients, a substantial negative association has been observed between HER2 expression and Lp(a) levels [163]. However, a recent study in a sizable general population cohort (*N* = 109,440) found no relationship among elevated Lp(a) levels and breast cancer risk, despite its role in predicting distant metastasis [164, 165].

Furthermore, compared to healthy controls, male patients with stage I–IV lung cancer had considerably higher Lp(a) levels, and these elevations strongly correlated with the lung cancer's stage, according to studies on the disease [166]. Remarkably, the research also revealed that Lp(a) might be involved in preventing lung cancers from growing both locally and metastatically [167]. Numerous other malignancies are also linked to an increase of higher Lp(a). For instance, Lp(a) levels are highly correlated with an elevated risk of prostate cancer, squamous cell carcinoma of the head and neck, and colorectal cancer and are markedly elevated in these patients [168, 169]. Considering the potential involvement of Lp(a) in the advancement and prognosis of multiple cancers, it is expected to be developed as a diagnostic or prognostic marker for cancer in the future. Further large-scale studies and mechanistic explorations will help to clarify the role of Lp(a) in different cancer types and provide a scientific basis for its clinical application.

With regard to the therapeutic aspects of Lp(a), it has been found that the therapeutic efficacy of immune checkpoint inhibitors (ICIs) can be enhanced by strategies that reduce Lp(a) levels (e.g., the use of statins or PCSK9 inhibitors) that enhance the response of tumor-specific cytotoxic T cells. For example, statins may enhance the efficacy of ICI by promoting immunogenic cell death in certain cancer types and attenuating checkpoint proteins that inhibit T cell function in the tumor microenvironment [170]. In addition, RNA therapies targeting Lp(a) and antisense oligonucleotide (ASO) therapies may enhance immune surveillance and anti-tumor responses in the context of cancer while reducing Lp(a) levels [171].

Overall, therapeutic strategies to reduce Lp(a) show great potential in cancer immunotherapy, although more studies are still needed to validate them. Future studies should further explore this area with the aim of providing new avenues for cancer treatment.

### Lipoproteins and other diseases

#### Diabetes mellitus (DM)

DM is a chronic metabolic condition characterized by absolute or relative insulin insufficiency and impaired insulin utilization [172]. Research indicates that HDL has some antidiabetic properties, as it can enhance insulin secretion, protect β cells, and reduce insulin resistance, this implies that HDL-based therapies could be beneficial, to a certain degree, for type 2 diabetes patients who do not respond well to antidiabetic medications [173]. Conversely, LDL-C levels show a linear positive correlation with diabetes risk [174]. Higher LDL-C levels are substantially linked to a higher incidence of diabetes in the general population, this association is especially evident in individuals with extremely low LDL-C levels who have not undergone lipid-lowering treatment [175, 176]. Additionally, increased LDL-C levels can worsen insulin resistance and vascular complications in diabetic patients, making LDL-C reduction an effective way to lower ASCVD event occurrence [177]. The number of individuals with type 2 diabetes is negatively correlated with Lp(a) levels, as indicated by certain studies [178]. However, high Lp(a) levels are linked to complications in diabetic patients, such as kidney failure, retinopathy, and neuropathy [179–181]. Furthermore, Lp(a) could potentially be a standalone indicator of recurrent cardiovascular events in patients with type 2 diabetes [182]. While the evaluation of Lp(a)-lowering treatments in diabetic patients remains limited, available research indicates that reducing Lp(a) may provide substantial benefits for cardiovascular outcomes. Even among non-diabetic individuals, Lp(a)-lowering therapies could influence the risk of developing diabetes, though the cardiovascular event prevention benefits may surpass the diabetes-related risks.

#### Chronic kidney disease (CKD)

CKD is characterized by kidney damage or impaired kidney function that persists for at least 3 months [183]. Increased LDL-C and lower HDL-C have been found to be closely associated with renal function [184]. HDL-C possesses anti-inflammatory and antioxidant properties that can alleviate kidney damage and delay the deterioration of kidney function [185]. Elevated LDL-C, by triggering local inflammatory responses and amplifying oxidative stress, exacerbates glomerulosclerosis and leads to further kidney function decline [186]. In summary, HDL-C is crucial in safeguarding kidney health, whereas excessive LDL-C accumulation worsens kidney damage. Recent research has shed more light on the connection between Lp(a) and kidney disease. There is evidence that higher Lp(a) levels are strongly associated with decreased estimated glomerular filtration rate, a phenomenon observed in both patients with mild renal impairment and those on hemodialysis for end-stage renal disease [187–190]. Furthermore, research has shown that Lp(a) levels in the renal vein are lower than those in the ascending aorta, indicating that elevated Lp(a) might be linked to reduced kidney clearance [191]. The cardiovascular risk in people with CKD is significantly elevated compared to the general public. and since CKD patients also exhibit elevated Lp(a) levels, pharmacological treatment to lower Lp(a) could be particularly beneficial for them. Nonetheless, more studies with larger sample sizes are needed to validate this.

#### Autoimmune diseases

Autoimmune diseases (AD) are defined as a group of conditions resulting from the loss of immune tolerance to self-antigens [192]. Research indicates that autoimmune diseases, including rheumatoid arthritis(RA) and systemic lupus erythematosus(SLE), are strongly linked to dyslipidemia, dyslipidemia can exacerbate abnormal inflammatory responses and heightened immune activity, which in turn impacts the quality of life and prognosis of AD patients [53]. Research investigating the relationship between Lp(a) and AD has revealed that RA and SLE patients exhibit significantly elevated serum Lp(a) levels [193, 194]. Moreover, studies indicate a strong association between the complement system in RA patients and various lipid molecules, including Lp(a) [195], and Lp(a) may also act as a reactant during active phases of RA [196]. Elevated Lp(a) levels are strongly correlated with kidney involvement in SLE patients, suggesting that Lp(a) may interact with immune system abnormalities to cause further kidney vascular damage [197].

Moreover, research has shown that in AD patients undergoing biologic therapy, different biologics have distinct impacts on Lp(a) levels. For instance, RA patients treated with tocilizumab show lower Lp(a) concentrations, while other biologics may have a smaller effect on Lp(a) levels [198, 199]. Given the typically elevated cardiovascular risk in AD patients, we hope that future studies will uncover whether variations in Lp(a) levels might differently affect cardiovascular risk in RA and other AD patients. Furthermore, evaluating whether Lp(a)-lowering therapies can enhance cardiovascular prognosis will be essential.

## Advances in research and clinical applications of lipoprotein reduction therapies

As noted earlier, abnormalities in lipoprotein levels are linked to the onset and progression of cardiovascular diseases, peripheral artery disease, neurological conditions, cancer, metabolic disorders, and autoimmune diseases. Currently, multiple treatment strategies exist to manage abnormal HDL-C and LDL-C levels, including statins, cholesterol absorption inhibitors (like ezetimibe), and PCSK9 inhibitors. These treatments are designed to lower the risk of ASCVD. However, while these therapies have been successful in reducing other lipoprotein levels, elevated Lp(a) continues to pose a unique and challenging cardiovascular risk factor to manage.

### Therapies for the reduction of common lipoproteins

Current treatment strategies for lowering lipoprotein levels primarily include lifestyle interventions, pharmacotherapy, and their combination, aimed at improving the lipid profile of patients and reducing the risk of cardiovascular disease. First, adjustments in lifestyle are regarded as the cornerstone for lowering lipoprotein levels. Research indicates that following a Mediterranean dietary pattern or a diet high in omega-3 fatty acids can significantly decrease LDL levels and simultaneously raise HDL levels [200, 201]. Furthermore, it has been demonstrated that frequent aerobic exercise, like brisk walking, swimming, or cycling, greatly enhances lipid metabolism [202]. Relevant studies have shown that exercise not only increases HDL levels, but also effectively reduces LDL and triglyceride (TG) levels [203].

Second, drug therapy has an important role in lowering lipoproteins. The most widely used class of medications is called statins, which significantly lower LDL levels by blocking HMG-CoA reductase, the enzyme that limits the synthesis of cholesterol. This lowers the chance of cardiovascular illnesses while also improving arterial endothelial function and having some anti-inflammatory properties [204, 205]. In addition to statins, bile acid binders (e.g., colestimide) lower cholesterol levels by binding to bile acids and promoting their excretion [206], while niacin works by reducing fat synthesis in the liver and increasing HDL levels [207]. In addition, cholesterol absorption inhibitors (e.g., ezetimibe) further reduce plasma LDL levels by inhibiting intestinal absorption of cholesterol [208].

In recent years, emerging targeted therapies such as PCSK9 inhibitors (e.g., alirocumab, evolocumab) have shown significant LDL-lowering effects, and are particularly suitable for high-risk patients or those who are poorly tolerant to statins [209, 210]. These drugs substantially reduce LDL levels in the blood by inhibiting the activity of the PCSK9 protein and enhancing LDL clearance by the liver. It has also been found that PCSK9 inhibitors may have cardiovascular protective effects and help improve cardiovascular prognosis [211].

Overall, general lipoprotein-lowering therapies combined with lifestyle modifications and medications form a multidimensional management strategy that helps to lower the chance of illness and improve the overall health of patients.

### Therapeutic approaches for reducing specific lipoproteins (Lp(a))

Lp(a) has been increasingly recognized as a risk factor for cardiovascular disease in recent years, and its potential link to non-cardiovascular diseases, including stroke, peripheral vascular disease, cancer, and diabetes, has also begun to attract attention. However, according to current expert consensus [10], in the absence of approved Lp(a)-lowering therapies, individuals with elevated Lp(a) should temporarily focus.

on early and aggressive management of other risk factors to reduce ASCVD risk. As a result, exploring pharmacological approaches to reduce Lp(a) levels has become a widely researched and highly prioritized field ( \* MERGEFORMAT Table 2).

**Table 2:** Mechanisms of action of different lipid-lowering therapies

Lipid-lowering therapy	Mechanism	Targeted Lipoproteins	Clinical effects
Statins	Inhibition of HMG-CoA reductase in the liver reduces cholesterol synthesis while indirectly increases LDL receptor expression and promotes LDL-C clearance, but the effect on Lp(a) is more limited	LDL-C	Statins significantly reduce LDL-C levels and are widely used in the prevention and treatment of cardiovascular disease, but have less effect on Lp(a) levels [199, 212]
Aspirin	Inhibits cyclooxygenase (COX), reduces platelet aggregation, and exerts antithrombotic effects. Aspirin partially attenuates the procoagulant and proinflammatory effects of Lp(a), but does not directly reduce Lp(a) levels	Lp(a)	(1) Aspirin lowers the risk of thrombotic events in people whose Lp(a) levels are high, but it doesn't change Lp(a) levels directly (2) Research backs up the use of aspirin to lower long-term ASCVD risk in people with elevated Lp(a) levels [213]
PCSK9 inhibitor	PCSK9 interacts to the LDL receptor on the surface of hepatocytes, contributing to their destruction. PCSK9 inhibitors promote the clearance of LDL-C and Lp(a) by blocking PCSK9 action and increasing the number of LDL receptors	LDL-C, Lp(a)	In several studies, such as the FOURIER and ODYSSEY trials, evolocumab and alirocumab have been shown to lower Lp(a) levels/LDL levels and reduce cardiovascular risk compared to placebo [214, 215]
Lipoprotein apheresis	Physically separating and removing LDL and Lp(a) from the blood significantly reduces the concentration of these lipoproteins in the blood	LDL-C, Lp(a)	Apheresis is especially useful for people who have an elevated chance of cardiovascular disease since it quickly and dramatically lowers Lp(a) levels. It is often used in patients who are difficult to control with medication. Physically separates and removes LDL and Lp(a) from the blood, significantly reducing the concentration of these lipoproteins in the blood [216]
Antisense Oligonucleotide (ASO)	ASO reduces Lp(a) synthesis by binding to the mRNA of the Lp(a) gene and blocking its transcription process	Lp(a)	Pelacarsen is a hepatocyte-mediated second-generation GalNAc-conjugated ASO drug. Phase I clinical study showed a dose-dependent significant reduction in Lp(a) in Pelacarsen patients compared to placebo [217]
RNA interference (RNAi) therapy	RNA interference prevents protein synthesis in Lp(a) by targeting Lp(a) mRNA and causing its degradation	Lp(a)	Inclisiran, an RNAi therapy, significantly reduces Lp(a) and LDL-C levels and demonstrates long-term stable lipid-lowering effects for sustained improvement in cardiovascular health [218]

#### Lipid-lowering agents

Statins, ezetimibe, and niacin are commonly used lipid-lowering medications in clinical practice. The former can boost the expression of hepatic LDL receptors, lowering circulating LDL-C levels [219], the latter two can reduce lipoprotein levels by blocking fat synthesis and absorption [212]. Despite the structural similarities between Lp(a) and LDL, statins do not appear to have the desired effect on lowering Lp(a) levels, and may even raise them [220, 221]. Similarly, the administration of ezetimibe, either alone or in conjunction with statins, did not produce any noteworthy impact on Lp(a) levels [222]. Only niacin resulted in a significant reduction in Lp(a) levels by decreasing apo(a) production [223].

Of note, because statins have been shown to significantly reduce cardiovascular events through a variety of alterations [224, 225], it is difficult to assess whether statins can affect cardiovascular outcome events through alterations in Lp(a). Additionally, we are curious whether adding Lp(a)-lowering therapies on top of statins could further decrease the occurrence of cardiovascular events. For instance, could PCSK9 inhibitors, either with or without statins, further reduce cardiovascular events by specifically lowering Lp(a) levels?

#### Aspirin

Aspirin, an antiplatelet agent, is the cornerstone for inhibiting platelet production and reducing the chance of serious cardiovascular complications after PCI [226]. Aspirin use has been found to lower the risk of distant ASCVD in people with elevated Lp(a) levels, according to an increasing body of research published in recent years [213, 227]. However, before a definitive recommendation can be made, the benefits of aspirin must be balanced against its inherent bleeding risks, and the 2022 Lp(a) expert consensus states that current data do not support the use of aspirin on the basis of lipoprotein(a) concentrations and that its benefit in individuals with significantly elevated Lp(a) is uncertain [10]. Additional high-quality randomized controlled trials are needed to further elucidate whether primary prevention with aspirin in patients with high Lp(a) is sufficient to reduce ASCVD events to justify the increased risk of bleeding in such patients.

#### PCSK9 inhibitors (monoclonal antibodies and siRNA)

As a prominent lipid-lowering target, PCSK9 protein has garnered increasing attention. Both the "old member" PCSK9 monoclonal antibodies and the new siRNA lipid-lowering drugs have been shown to enhance lipid management to control the risk of cardiovascular disease [214]. While the mechanism by which PCSK9 inhibitors lower LDL-C is clearly defined, their potential effect in reducing cardiovascular risk by lowering Lp(a) still needs to be explored further. Current studies (such as FOURIER and ODYSSEY) indicate that evolocumab and alirocumab have statistically significant effects on lowering Lp(a) levels [228, 229]. The siRNA-based lipid-lowering drug inclirisan also demonstrates a reduction in Lp(a) levels, which has been confirmed in ORION 10 and ORION 11 [215]. However, the evidence provided by these studies is only suggestive, and has yet to definitively prove the direct association between the reduction of Lp(a) by PCSK9 inhibitors and cardiovascular risk. In addition, the results of these trials primarily focus on secondary prevention and exhibit enhanced advantages in patient cohorts with markedly elevated Lp(a) levels; consequently, the generalizability of these findings to a broader population remains to be established [228, 229]. Despite the absence of a notable difference in Lp(a) reduction attributable to statins, evidence indicates that evolocumab enhances Lp(a) catabolism in conjunction with statins by markedly up-regulating the LDL receptor, thereby improving Lp(a) particle clearance [230]. Nevertheless, other research has indicated that the LDL receptor does not significantly contribute to this process [231], so more clinical trials are needed to explore whether lowering Lp(a) concentrations in addition to other lipid-lowering drugs, especially statins, is more beneficial in terms of cardiovascular risk. In addition, future studies may focus on stratifying according to baseline apoB levels to assess the heterogeneity of Lp(a)-reduced outcomes to more fully understand its role in cardiovascular prevention. We look forward to future studies bringing us new findings and insights(NCT05952869,NCT05888103,NCT04873934).

#### Lipoprotein monoculture, ASO and RNA interference therapy

As previously stated, most lipid-lowering drugs reduce Lp(a) levels non-specifically to mitigate ASCVD risk. Currently, here are no approved medications that specifically target the reduction of Lp(a) levels. The only FDA-approved treatment for lowering Lp(a) levels to date is lipoprotein apheresis, which can significantly lower Lp(a) concentrations, particularly in those with extremely high Lp(a) levels. However, although it has shown significant effectiveness in reducing Lp(a) levels, lipoprotein apheresis has not been widely accepted as a standard method for primary or secondary prevention of cardiovascular diseases. Moreover, because of its high cost, time-consuming process, and difficulty in testing through randomized clinical trials, the application of this therapy is currently limited [216]. In this context, selective Lp(a) reduction therapies based on apo(a) antisense oligonucleotides (ASOs) and siRNA-based technologies have been developed. The former seeks to decrease plasma levels of Lp(a) by causing apo(a) mRNA degradation, blocking apo(a) synthesis in the liver, and reducing Lp(a) secretion in particular [219], while the latter accomplishes the same goal by reducing Lp(a) levels through inhibiting the translation of the LPA gene's mRNA in hepatocytes and mutating the causing gene [232].

Pelacarsen is a hepatocyte-mediated second-generation GalNAc-conjugated ASO drug [233]. Phase I clinical trial indicated a dose-dependent substantial reduction in Lp(a) in Pelacarsen patients compared to placebo [234]. Phase III trials are presently underway to evaluate the benefits of Pelacarsen over placebo in reducing the chance of long-term cardiovascular incidents in individuals who have high levels of Lp(a), Let's wait and see.

In addition, targeted therapeutic agents utilizing small interfering RNA technology are being evaluated. Olpasiran (AMG890) has shown a dose-dependent significant reduction in Lp(a) concentrations [235], OCEAN(a)-Outcome (NCT05581303) is an ongoing phase III clinical trial designed to study its long-term effects on MACE. Recently, many advances have been made in siRNA therapeutics targeting Lp(a). Silence Therapeutics, Inc. announced new data from its Phase I clinical trial of zerlasiran (formerly SLN360), an siRNA therapeutic targeting Lp(a), which showed that within 90 days of two subcutaneous administrations, patients experienced a significant reduction in Lp(a) levels compared to baseline, which was as much as 99% lower and well tolerated [236]. At the American Heart Association Scientific Sessions 2023, Eli Lilly and Company announced positive results from the first human trial of its siRNA therapy lepodisiran, the results showed that a single injection of lepodisiran resulted in well-tolerated and dose-dependent, long-term sustained reductions in serum Lp(a) levels [237], And its large-scale Phase II study is underway.

Notably, in addition to subcutaneous injections, the first oral Lp(a)-reducing small molecule, Muvalaplin, has also recently achieved a breakthrough, with a Phase I clinical study demonstrating that daily oral doses of Muvalaplin over a 14-day period reduced blood Lp(a) levels by up to 65 per cent [238]. The effects of these new drugs on long-term cardiovascular outcomes are unclear, and multiple drugs are being studied. These emerging therapies, aimed at specifically reducing Lp(a) levels, promise to usher in a new era in the quest for Lp(a) [34].

## Conclusions and outlook

Lipoproteins and apolipoproteins play crucial roles in health and a variety of diseases, particularly in the cardiovascular disease field. Multiple studies have clearly shown that abnormal lipoprotein levels are strongly linked to atherosclerotic cardiovascular disease and are also important in metabolic, neurological, and oncological diseases. Despite the great success of traditional lipid-lowering treatments, such as PCSK9 inhibitors and statins, in lowering LDL-C and the chance of atherosclerotic cardiovascular disease, the distinct pathogenic mechanism of Lp(a) remains a major clinical management challenge.

Robust evidence suggests that Lp(a) is a highly anticipated emerging target for intervention, increasing cardiovascular risk by promoting atherosclerosis and inflammation. Clinical research have revealed a robust and continuous link between Lp(a) levels and cardiovascular illnesses such as coronary artery disease and calcific aortic stenosis. However, it is crucial to emphasize that because of insufficient large-scale randomized clinical trials, the significance of Lp(a) in cardiovascular disease and other clinically relevant disorders (such as atrial fibrillation, heart failure, stroke, cancer, etc.) still requires further confirmation. We anticipate that more high-quality clinical trials will offer a more complete understanding of the relationship between high Lp(a) levels and these diseases, potentially offering new secondary prevention options for high-risk populations. Moreover, existing lipid-lowering therapies have only a minimal impact on Lp(a) levels, and their effects on long-term cardiovascular outcomes are not well defined. While there are no FDA-approved therapies targeting Lp(a) reduction, sufficient evidence supports ASO and siRNA therapies for specifically lowering Lp(a) concentrations (albeit still in early-stage trials). Although definitive evidence is still lacking, it remains to be seen whether these emerging therapies, in addition to lowering Lp(a) levels, will also positively affect cardiovascular and other diseases. We look forward to these ongoing clinical trials bringing us a promising answer.

Clinically, Lp(a) screening and early management should be incorporated into the evaluation of high-risk individuals, particularly those whose cardiovascular disease risk is not fully explained by traditional risk factors. Considering that the AHA/ACC guidelines have recognized Lp(a) as a key independent indicator of ASCVD risk, future studies should aim to enhance screening strategies for earlier identification of high-risk individuals and personalized intervention. In conclusion, despite notable advancements in therapies to lower LDL-C and other lipoprotein levels, Lp(a) remains an insufficiently explored aspect of cardiovascular disease management. By deepening our understanding of its pathogenic mechanisms and developing novel clinical therapies, the future may hold promising improvements in the prevention and treatment of cardiovascular diseases globally.

## Data Availability

Not applicable.
